# Editorials

**Published:** 1899-01

**Authors:** 


### EDITORIAL.

### GREATER NEW YORK IN 1899.

After the longest period of absence from the Metropolis, since its birth, more than thirty years ago, the National Association returns to Greater New York, stronger in power, greater in number than ever before. It meets again in this great centre of veterinary learning for the first time under its new name, and as the American Veterinary Medical Association it will receive the warmest welcome it is possible to give by the very large number of earnest, aggressive veterinarians who centre at and adjacent to this great field of veterinary science. Since its last meeting there the Association has lost a large number of its members in this territory, who found it impossible to attend our meetings, and thereby lost interest in its work. These all should come back in "'99," and bring many others whose names should be enrolled upon the roll of honor that makes up our membership and stands for every good and wise movement in the advancement of our profession. The quickened zeal of those engaged in teaching in the Empire State should receive an earnest expression of appreciation by those who will gather there to promote the Association's power, warm its influence, and extend its strong arms around a still greater number who need its support and assistance.

---

### NOW FOR ARMY RECOGNITION.

The hour for striking one united blow for rank in our army is at hand, and the battle so long waged for recognition will be won if there is a united and single effort made from all over the land. Before another issue of the Journal reaches our readers our fate in this direction will no doubt be sealed. Every veterinarian has been reached whose address was known, and now we make the final appeal to every one who has not taken up his part of the work. To your Senators and Congressmen you must go for aid in this direction, and to them appeal for assistance in according us in our army reorganization that which we have long deserved, that which has been wrongfully denied us, and for which our government has paid severely in many ways. Your influence, your outspoken support, the assistance of your friends, may be the final spoke in the wheel to set going the vehicle of progress along this line. Many State organizations are active in work; the Army Legislative Committee of the American Veterinary Medical Association are aggressive and determined, but much will be lost without your personal aid and support, and this last appeal is made to you now by those who fully appreciate the critical hour at hand. Your appeal, your friends' appeal, your clients' assistance, your association's efforts will all count for much in the final struggle. Make the place of the army veterinarian worthy of the best of our profession. Make it a fitting place of honor for your sons and your successors; make it a place of joy for those who have given the best years of their lives in the country's service.

---

### AN EXCELLENT APPOINTMENT.

THE Veterinary Department of the Agricultural College of Iowa in the selection of Dr. John J. Repp, of Pennsylvania, for the chair of Pathology and Therapeutics and Veterinarian to the Experiment Station, have made an excellent choice. Dr. Repp is a graduate of the Veterinary Department of the University of Pennsylvania, a member of the American and Pennsylvania Veterinary Medical Associations. Since his graduation he has been associated with the work of the Pennsylvania Livestock Sanitary Board. As a licensed practitioner of the Keystone State, he obtained the highest average ever given by the Board. Earnest, thorough, and conservative in all his work, a sincere devotee to his profession and all its future hopes and aims, he is sure to bring to his new field of labor the best results that are sure to flow from a well-equipped and well-balanced mind and judgment.

---

Many friends among the profession all over the land will learn with much regret, and voice their sincere sympathy, that our esteemed colleague, Dr. Thomas B. Rayner, of Philadelphia, who was afflicted some two years ago by the loss of a son and prospective successor, has again felt the sting of death in the loss of an estimable daughter. Since writing the above, the death of a brother, Dr. Harry B. Rayner, has been added to the load of sorrow afflicting our esteemed fellow-worker.

---

THE Alumni of the Veterinary Department of McGill University will have a very enjoyable reunion at the old College Hall in

Montreal on February 4th next, and the JOURNAL desires to extend its best wishes for the success of the movement and the school that has done so much for higher veterinary education.

